# What Should Be Next in Order?

**Published:** 1901-06

**Authors:** 


### Editorial.

### WHAT SHOULD BE NEXT IN ORDER?

EACH day, week, month, and year brings its changeable duties, and he will be a wise man who can regulate in advance the direction of the life-current or where it will land the individual, the association, or the community. The world at large is full of surprises. One year the Autocrat of all the Russias will call the nations together to formulate plans for the preservation of the peace of the world, and before that world has had time to digest the contents of the signed contract of the Powers, war has become the universal thought and the universal practice. What is true of nations is true of the individual, and it is this uncertainty that is always attractive if not always standing for true progress.

The intellectual world, of which dentistry should form a part, is subject to the same surprising fluctuations. This should be expected, for, after all, mind is the motive power behind all these movements, and the fact that nations are unrestful presupposes an internal mental force that will change the character of all social conditions. While the upheavals are not always for the good of peoples, they invariably indicate a mental activity that must eventually end somewhere and somehow in the uplifting of the race and the betterment of those civilizing tendencies that are the marking stones of progress.

These reflections belong particularly to this period in dental education, as well as to dental work in general. It would be an anomaly to have dentistry quietly stranded while the torrent is rushing through the greater world to more enlarged aspirations and more notable results. This has not heretofore been the condition of this the new-born of the professions. Its vigorous life has kept it in the van of progress, but to remain there means constant vigilance and a constant subjugation of selfish aims.

It must be apparent to all reflecting minds that the educator is the leader of the people. That the school-master has been relegated to a somewhat inferior position is true, but the world has not yet come to a period when it is capable of analyzing the forces that move it onward in a spiritual and moral sense. When that time arrives the teacher will occupy, as he or she should do now, the loftiest pinnacle in the world's regard, and it is strange that it is not recognized that behind kings and emperors, power and dominion, stands always the teacher guiding and directing the unseen, oftentimes unfelt, but always the potent force that directs the world in its onward march for good or evil. The teachers of every age have been nailed to a cross, and the cruel death of the great Master-Teacher of the ages simply typifies the eternal injustice accorded to these the saviours of the race, who combine within themselves the uplifting power that has universally tended to a more refined civilization.

The application of these truisms is not difficult. We need a higher appreciation of those who have been leading the dental hosts than has been accorded them. The dental college teacher is simply following in the wake of his clan in other fields of work. He probably would not have it different, so far as his individuality is concerned, but that which does trouble him is the indifference, the almost contempt, which seems to be felt for his efforts to raise the dental profession to a higher level. The colleges, and by that is meant the teachers, for bricks and mortar are soulless, are the choice mark for the man on the retrograde. They are to him an everlasting sign of his own weakness, and hence his antagonism. In his personality are represented the retarding forces that would, if they could, relegate civilization to aboriginal conditions, the age of the implements of stone.

The teachers of this day in dentistry ask that the profession continue to move steadily forward to higher levels. They have not urged this in vain, for much has been gained, and, while progress has been slow, there has been no backward step taken. It is, however, a truth that as a body we seem to have been turned out of the deep current and are floating in shallow waters. This may not be apparent to all, but to the writer there are unmistakable signs that the good ship is not moving as rapidly as would seem desirable. Is this the fault of the crew or of uncontrollable circumstances? To drop the figure, are we doing all that is possible to meet changing conditions? It would seem that we have not been doing all that is possible, for the fruits by which we are to be known are not satisfactory. The teachers may be there, but if so, there is something wrong in the teaching.

The lamentable exhibit at Washington recently of only two passing the board out of fourteen examined, while it may not prove this to be true, goes very far to demonstrate that there is a defect which needs remedial aid, and that very speedily. It is possibly true that the army board may be partially to blame in this instance, for commissions are very apt to go wrong even when most intelligently constituted, and it may be considered an open question with this one; but, allowing for all this, it is quite evident that the unlooked-for result means for the dental colleges a more stringent method, if not a new standard of training.

The Surgeon-General possibly unwittingly hit the nail directly when he stated that he wanted practical men as contract dental surgeons. It is just here where the dental profession is standing to-day. It needs practical men.

The early work of dentistry developed this class, but failed to enlarge the intellectual horizon. The subsequent periods have sent the minds of the students into a region of scientific knowledge and unfitted them, in a large degree, to follow in the footsteps of the Fathers and become practical men. At a recent meeting of one of our societies this dividing line between past and present was singularly evident in a discussion of why teeth were checked, cracked in crown-work. The young men had their theory, and charged it on the porcelain; the old men retorted that checking and cracking was almost unknown in the practical days of silver and gold plates. The contention typified the two ages and could not be harmonized.

The question is now, How can these two conditions, seemingly so antagonistic, be brought into harmony? We have talked about it, and the two teaching associations have spent laborious hours in attempts to solve the problem, but it practically remains in its present unsatisfactory state.

It is not the purpose of this article to point out the way. The problem is too deep, too far reaching, to be settled in the limited space of an editorial, but the subject-matter must be considered, and that by many minds, in the near future. How may we combine the practical with the theoretical without sacrificing either? We do not want rivers of words, but we do need, sadly need, practical suggestions that will lead us out of the almost pitiable situation in which we are at present placed. We need teachers, not mere men. We must have those capable of imparting the knowledge they may possess. We should have more time, and the National Association of Faculties will be negligent in its duty if it fails at its next meeting to add another year to the course; indeed, it will amount almost to a crime to stop progress. Practical men cannot be made as things now are. Why, then, hesitate? While it would be well to advance the entrance requirement, if anything must be sacrificed let it be that, but give the colleges at least time to teach. The Association of Dental Faculties has the power to effect this. At its next meeting the question will come before it. Let it not be said that the members left Milwaukee with the reflection that they failed in a manifest duty, and by thus doing placed the accomplishment of those things that make for progress in our profession to a day remote in the future. Are we ready to direct our ship from sluggish waters to the broad current of the new stream, so clearly manifest in the not far distant future, and which can be realized in part in the present if we are true to ourselves, true to our profession, and true to that high position which belongs to us as teachers in this important work?

